# Haematuria on the Spanish Registry of Glomerulonephritis

**DOI:** 10.1038/srep19732

**Published:** 2016-01-28

**Authors:** Claudia Yuste, Francisco Rivera, Juan Antonio Moreno, Juan Manuel López-Gómez

**Affiliations:** 1 Gregorio Marañón Hospital, Madrid, Spain; 2 Ciudad Real Hospital, Ciudad Renal, Spain; 3 Renal,Vascular and Diabetes Research Lab. IIS-Fundación Jiménez Díaz. Autonóma University, Madrid, Spain

## Abstract

Recent studies suggest a pathogenic role for glomerular haematuria among renal function. However, there is no data on the prevalence of haematuria from a large renal biopsy registry. We analysed the prevalence of gross (GH) and microscopic (mH) haematuria in 19,895 patients that underwent native renal biopsies from the Spanish Registry of Glomerulonephritis. Haematuria’s overall incidence was 63% (GH 8.6% and mH 55.1%), being more frequent in males (64.7% vs. 62.4%). GH was more prevalent in patients <18 years (21.3% vs. 7.7%). The commonest clinical presentation associated with GH was acute kidney injury (31.5%) and IgA Nephropathy (IgAN) (33.6%) was the most frequent histological finding. GH patients showed a significantly (p < 0.05) lower eGFR and proteinuria levels as compared with patients with mH and without haematuria. Moreover, mH was more prevalent in adults (56.3%). Nephrotic syndrome was the commonest clinical presentation in mH patients (32.2%) and IgAN (18.5%) the most frequent histological finding. In conclusion, haematuria, is a frequent urinalysis finding in patients underwent native renal biopsy. The most frequent histological finding in both GH and mH is IgAN. Whereas, GH is more frequent in young males with acute kidney injury, mH is commoner among adults with nephrotic syndrome.

Haematuria is defined as the presence of more than 2–5 red blood cells (RBCs) per high-power field in the urine^1^. Haematuria is called macroscopic or gross haematuria (GH) when there is massive presence of RBCs in the urine being identified by the naked eye, whereas microscopic haematuria (mH) can only be detected by microscopically examination of urine sediment. The presence of dysmorphic RBCs in the urine distinguish the glomerular origin of haematuria^2^. Glomerular haematuria is associated with glomerular filtration barrier (GFB) dysfunction or damage, as reported in renal diseases associated with abnormalities in GFB components or in inflammatory diseases where proteolytic enzymes released by inflammatory cells may degrade GFB components, thus leading to a more fragile GFB that is more susceptible to rupture, as seen in primary glomerulonephritis, autoimmune diseases, or infections^3^.

Haematuria has been used as a marker of renal disease activity and it is considered as one of the hallmarks of some glomerular diseases, such as IgA Nephropathy (IgAN), Alport syndrome (AS), Endocapillary GN, Membranoproliferative (MPGN) and Crescentic GN, but rather unusual in other nephropathies including Minimal Change Disease (MCD), Focal and Segmental Glomerulosclerosis (FSGS) and Membranous Nephropathy (MN)^4^,^5^,^6^. Although glomerular haematuria has been traditionally considered a benign condition, it has been recently associated with a negative renal function outcome in the short^7^,^8^ and long-term^9^,^10^. Therefore is however no robust data on the prevalence of haematuria among patients with renal dysfunction that have undergone renal biopsy, or its relationship with other glomerular injury markers, such as proteinuria and serum creatinine.

In this study we aimed to investigate the prevalence of gross and microscopic haematuria and its association with clinical and histological findings in 19,895 patients collected in the Spanish Registry of Glomerulonephritis between 1994 and 2013.

## Results

The clinical characteristics of the 19,895 patients who underwent native renal biopsy are shown on Table 1. Haematuria was present in 12,683 patients (63.7%), of whom 1,717 patients presented with GH (8.6%) and 10,966 patients with mH (55.1%). Patients with GH were significantly younger than the ones with mH and those without haematuria (p < 0.05). Haematuria was more frequent in males than in females (64.7% vs. 62.4%, p < 0.05). GH patients presented significant higher serum creatinine concentration and lower eGFR and proteinuria levels than patients with mH or NH. Hypertension was found in 53.5% patients, being significantly more frequent in mH as compared with GH and NH patients.

### Evolution of biopsies over 2 decades

The total number of biopsies fluctuated during the 20 years analysed in our study (Supplementary Table 1). The prevalence of haematuria decreased with time, due to a decrease presence in the number of mH cases. However, mH remained the commonest urinalysis finding throughout. We observed a progressive increase in age of the patients who underwent renal biopsy, from 44.2 ± 20.2 years in the beginning of the nineties to 52.0 ± 18.4 years in the last period. This aging was especially striking in GH patients (from 38.6 ± 23.4 years to 52.8 ± 19.9 years, p < 0.05). The eGFR at the time of the biopsy decreased progressively during the study (from 53.7 ± 38.4 to 48.8 ± 36.2 ml/min), being especially noticeable in GH cohort (from 55.9 ± 47.9 to 37.9 ± 34.3 ml/min), while the eGFR at time of biopsy in NH group remained stable. The level of proteinuria fluctuated during the 5 time-points of the study and in relation of the presence of haematuria, it increased in GH patients, but remained stable in both mH and NH patients.

### Renal syndromes and urinalysis finding

**Figure 1:**
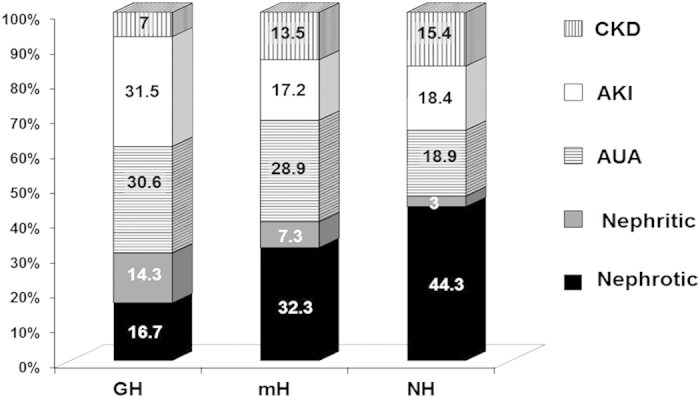
Indication for native renal biopsy according with urinalysis. GH, Gross Haematuria; mH, Microscopic Haematuria; NH, Non Haematuria; CKD, Chronic Kidney Disease; AKI, Acute Kidney Injury; AUA, Asymptomatic Urinary Abnormalities.

The main renal syndrome at the time of the renal biopsy in the whole Registry was nephrotic syndrome (35.3%), followed by AUA (21.3%), and AKI (18.9%). The most common renal syndrome among GH patients was AKI, while nephrotic syndrome was the most frequent syndrome in mH and NH patients (Fig. 1).

**Figure 2:**
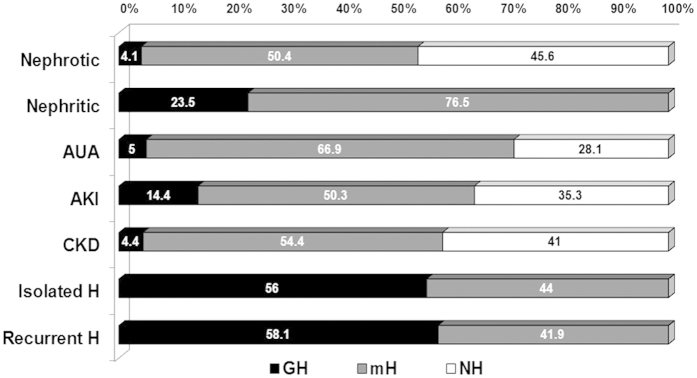
Urinalysis finding according with the main renal syndrome. GH, Gross Haematuria; mH, Microscopic Haematuria; NH, Non Haematuria; CKD, Chronic Kidney Disease; AKI, Acute Kidney Injury; AUA, Asymptomatic Urinary Abnormalities; Isolated H, isolated haematuria; Recurrent H, recurrent haematuria.

On the other hand, haematuria was found in the 54.1% of patients with nephrotic syndrome, mainly mH (Fig. 2). Similarly, mH was also significantly more frequent than GH in the patients with Nephritic syndrome. Almost 65% of AKI patients presented with haematuria, mainly mH. Additionally, 58.8% of CKD patients had haematuria. (Fig. 2).

### Urinalysis findings according to gender and age groups

**Figure 3:**
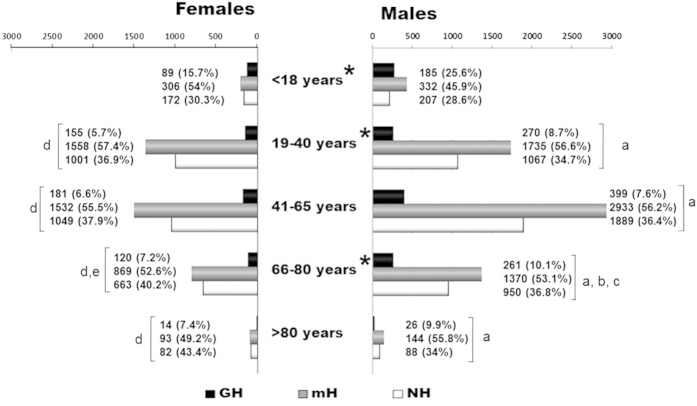
Urinalysis findings according gender and age groups. GH, Gross Haematuria; mH, Microscopic Haematuria; NH, Non Haematuria. Where * represents statistically significant differences on the urinalysis findings between genders in the same age group. Where ^a^ represents the differences with the <18 year-old male group, ^b^ differences with the 19–40 year-old male group and ^c^ differences with the 41–65 year-old male group. Where ^d^ represents differences with the <18 year-old female group and ^e^ differences with the 19–40 year-old female group.

Microscopic haematuria was the most common urinalysis finding, independently of age or gender (Fig. 3). Haematuria was more frequent in young patients (70.7%, <18 years vs. 63.1%, >18 years), especially in the GH group (21.3%, <18 years vs. 7.7%, >18 years). The urinalysis findings in the childhood and adolescent cohort were significantly different from the over 18 years of age (GH 20.7%/mH 50%/NH 29.3% vs. GH 8%/mH 54.6%/NH 37.4%, respectively). Elderly males (66–80 years) presented a higher GH prevalence compared with young adults (19–40 years) and adults (41–65 years) (10.1% vs. 8.7% and 7.6%, p < 0.05, respectively). Similarly, elderly women also presented higher prevalence of GH than young adult females (GH 7.2% vs. 5.7%). Patients aged more than 80 years presented similar urinalysis findings than young adults and adults in both genders (Fig. 3).

### Histopathological findings according to degreed of haematuria

IgAN was the most common histological diagnosis in our study, followed by Crescentic GN, MN, Lupus Nephritis (LN) and FSGS (Table 2). IgAN was the most common histological diagnosis in GH patients, followed by Crescentic GN and MPGN, Mesangial non IgA and LN. The principal diagnosis in mH cohort was also IgAN, closely followed by Crescentic GN, LN and MN. On the other hand, the most frequent histological finding in NH patients was MN, followed by MCD, LN and FSGS.

**Figure 4:**
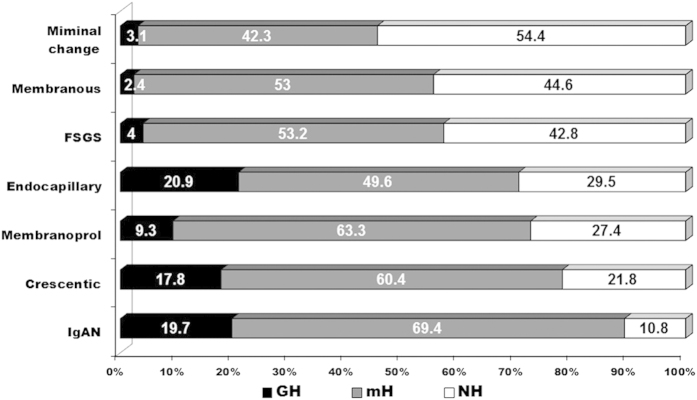
Primary Glomerulonephritis according with its urinalysis presentation. GH, Gross Haematuria; mH, Microscopic Haematuria; NH, Non Haematuria; IgAN, IgA Nephropathy; FSGS, focal and segmental glomerulosclerosis.

When we analysed the presence of haematuria in primary glomerulonephritis, we found that almost 90% of all IgAN patients had haematuria, usually mH (Fig. 4). Similarly, haematuria was present in almost 80% of Crescentic GN, 72.6% of MPGN and 70% of Endocapillary GN (Fig. 4).

After working out the percentage of GH vs mH for each singular aetiology we observed that the highest GH percentages were among patients with anti-glomerular basement membrane (anti-GBM) disease, type 2 Crescentic GN and Endocapillary GN; whereas the highest mH percentages were associated with IgAN, ANCA-vasculitis and MPGN (Table 3). Finally, haematuria was presented in 41% of both acute and chronic tubulointerstitial nephritis.

Logistic regression reported that youth, male gender, low proteinuria and impaired eGFR were independent risk factors associated to both GH and mH. After stratification of patients according to age, our results show that male gender, low proteinuria and impaired eGFR were independent risk factors for GH, especially in patients older than 18 years. However, male gender was only an independent risk factor for mH in patients older than 18 years. Low proteinuria and impaired eGFR were associated with mH disregarding the age (Table 4).

## Discussion

We found a high incidence of glomerular haematuria in patients who underwent native renal biopsy. The majority presented with microscopic haematuria. The gross haematuria cohort included mostly males, during childhood and adolescence, and this was usually associated to AKI. Whereas the incidence of microhaematuria was higher in adults presenting with nephrotic syndrome.

Our findings regarding clinical presentation and histological diagnosis in biopsy-proven renal diseases are consistent with data reported from other European countries^11^,^12^,^13^. The most common indication for renal biopsy worldwide, as well as in our cohort, is nephrotic syndrome^14^,^15^,^16^,^17^. Likewise, we found that IgAN is the most common primary glomerulonephritis. This is in agreement with data from Western Europe^11^,^12^,^18^. Though studies from Eastern Europe^19^ and sub-Saharan Africa^16^ found MPGN to be most frequent diagnosis of glomerulonephritis. This finding is thought to be related to socioeconomic status.

Although is commonly accepted that glomerular haematuria indicates GFB dysfunction, its usefulness as glomerular injury marker is not properly established and so it is not formally included as a biopsy criterion in the KDIGO Clinical Practice Guidelines^20^. Similarly, although it has been suggested that early identification of glomerular disease optimizes treatment and may be beneficial on early CKD stages there is no consensus on whether biopsy should be performed in patients with haematuric glomerular diseases^21^,^22^. Recent evidences reported that persistent isolated haematuria increased 18-fold the risk of ESRD^9^, mainly due to primary glomerular diseases, pointing that persistent mH could be an early feature of glomerulonephritis. However, Gutierrez *et al.* suggested that biopsy assessment would be an unnecessary procedure in IgAN patients with minimal proteinuria and haematuria based on the good prognosis of the condition^23^. It is important however, to note that almost 33% of the IgAN cohort with GH bouts did not recover basal renal function after an AKI episode, inferring that renal biopsy should be only recommended in these IgAN patients^7^. On the other hand, renal biopsy is still mandatory in at least one member of a family affected with familial haematuria, whereas molecular testing may be performed in the remaining affected members^22^. In our opinion, and based on the high prevalence of haematuria in glomerular diseases found in our study, every patient with persistent mH should be carefully investigated to rule out primary and secondary glomerulonephritis. Moreover, renal biopsy should be performed a whenever patients present with haematuria associated with AKI, an episode of GH, heavy proteinuria^23^ or RBCs cast.

The real incidence of haematuria in the general population is uncertain, ranging from 0.18 to 16.1% in adults^24^,^25^ and from 0.03–1.5% in children^9^,^26^,^27^,^28^. These wide ranges could be explained by differences in gender, age, race, socioeconomic status, and haematuria´s screening method. A higher prevalence of haematuria in biopsy registry studies has been reported. The occurrence of glomerular haematuria was 75.8% in 4004 Czech patients that underwent renal biopsy, where GH and mH were found in 9.2% and 65.9% of patients, respectively^29^. We observed a lower prevalence of haematuria (63.7%), and this may account for the lower percentage of mH (55.5%) in our cohort. These disparities could be explained by the fact that the Czech cohort was 10 years younger than ours (37.9 vs. 48.3 years) where one in four patients were younger than 18 years (vs. one in twenty-five in our cohort). In our study, haematuria was more frequent in male patients <18 years, where GH ratio was 1.6:1 compared with the 0.85:1 for mH. In agreement with our data, bouts of GH are more frequent in children with IgAN and Endocapillary GN^30^,^31^. Notably, almost all of the 75% of children diagnosed with IgAN presented GH bouts in the first years of the disease^32^. The GH rebound observed in our elderly population (66–80 years), could be attributed to the striking Crescentic GN incidence in this group^33^,^34^.

Furthermore, the occurrence of haematuria, mainly mH, decreased progressively over the 20 years of our study. In line with this reduction, we observed a progressive decrease in the eGFR and higher age of patients undergoing renal biopsy. This indicates a more conservative renal biopsy criteria in the last years, hence avoiding to carry out biopsies the young population without significant renal impairment or almost nephrotic proteinuria in spite of their haematuria.

Although the genesis of haematuria remains unknown, it has been proposed that mH could be secondary to RBCs passage through the GFB between the endothelial cell gaps^35^,^36^. This mechanism has been observed in both non-inflammatory renal diseases, such thin basement membrane nephropathy (TBMN)^35^, as well as in inflammatory renal diseases, including MCD^37^, LN^38^, MN^39^, and focal^40^, and mild proliferative GN^41^. It has been suggested that an aggression over the GFB, irrespective of the cause, results in a weak point where the GFB could be easily open by even simple mechanical factors, as high intravascular pressure^42^. This hypothesis could explain the common association between nephrotic syndrome and haematuria observed in over the 55–66% of the patients^29^. On the other hand, breaks or holes in the glomerular capillary wall had been reported in necrotizing or crescentic forms of inflammatory GN^35^, presenting with GH. It has also been observed that during GH bouts in Alport syndrome patients there is a repaired glomerular basement membrane focal ruptures^43^. This considerable histological GFB injury could explain the high prevalence of GH in Anti-GBM disease and Endocapillary GN, as well as the high percentage of AKI patients presenting GH (31.5%), reported in our study. AKI is a common complication of severe GH^44^, with an incidence of 30% in IgAN patients^45^. After decades of considering GH bouts as an innocuous feature of glomerular diseases the negative implications of GH over renal outcome have been reported in IgAN^46^, AS^22^, and C3 Glomerulopathy^47^. These studies suggest that a better understanding of the role of haematuria on renal function outcome is necessary.

Haematuria has been classically considered as a hallmark of glomerular diseases, such as IgAN, Endocapillary, MPGN and Crescentic GN, but rather exceptional for some others, including MCD, FSGS and MN. Nevertheless, systematic reviews usually overlook to report the prevalence of haematuria^6^,^48^, even in IgAN^30^. Haematuria is the distinctive IgAN feature, usually presenting as persistent isolated mH, with occasional bouts of GH associated to infections. Haematuria´s prevalence in IgAN has been estimated at over 90% in patients with renal impairment, and almost 75% in patients with preserved renal function^49^. In agreement with our findings, haematuria is a usual feature of Endocapillary GN^29^,^50^ Crescentic GN^31^ and MPGN^51^. Our results also confirm previous reports^4^,^52^ of an unexpectedly high haematuria prevalence in traditionally considered non-haematuric glomerulonephritis MN, FSGS and MCD, pointing that the GFB injury that results in a heavy urinary protein leak, could as well allow RBCs egression.

The prevalence of haematuria in secondary glomerulopathies has not been formally reported. RBCs cast, but not haematuria, is a criterion for the diagnosis of systemic lupus erythematosus^53^, although haematuria is a common urinalysis finding^54^. Actually, isolated haematuria has been proposed as a marker of lupus activity^55^, associated with rapidly progressive glomerulonephritis^54^,^56^. However, mH is not a good biomarker of LN treatment response^57^ or renal outcome^58^.

Interestingly, we also found a high prevalence of haematuria (around 40%) in tubulointerstitial diseases, such as ATIN and CTIN. Similarly, Fogazzi *et al.* reported the presence of haematuria in the 47.6% of ATIN patients and a high prevalence of RBCs casts in the urinalysis of these patients^59^. It has been proposed that the presence of RBCs in the urine may be a consequence of the interstitial inflammation, which would result in a disruption of interstitial blood vessels and further RBCs extravasation through gaps of the tubular membrane^59^. The same mechanism had been proposed to explain the haematuria of sickle cell disease^60^.

Haematuria seem to be more frequent in males^22^,^30^ with a reported 2:1 ratio in young males^9^. However, haematuria is more frequent in TBMN females^29^ and some immune-mediated GN as LN^12^. Although there are some inherited nephropathies with X-linked transmission, such as X-linked Alport syndrome and Fabry disease, it is unknown why haematuria is more frequent in males. It is also unknown why males tend to progress faster than haematuric females in Alport Syndrome^47^, TBMN, C3 glomerulopathy^22^ and Fabry disease, but not in IgAN. In addition, we found that GH was more prevalent in males.

To our knowledge, this is the largest cohort study published on the prevalence of haematuria. It included renal biopsies, clinical presentation and histological diagnosis of haematuria over 20-year period. The main limitation of our study is related to the inherent heterogeneity of the cases recorded in a large multicentre renal biopsy registry, like the misclassification of some diseases that required electron microscopy diagnosis as TBMN and C3 glomerulonephritis.

In conclusion, haematuria is a highly accessible glomerular injury marker usually present among patients with renal impairment justifying the need for native renal biopsy. The most frequent histological diagnosis in both GH and mH were IgAN and Crescentic GN. Gross haematuria is more frequent in young males and is usually associated to AKI, whereas the incidence of microhaematuria is higher in adults with nephrotic syndrome.

## Material and Methods

A total 21,240 first native renal biopsies from 156 different hospitals were included in this retrospective study of Spanish Registry of Glomerulonephritis, covering a period 1994 and 2013^33^,^61^. We excluded those biopsies where urinalysis data was not recorded, so we finally studied 19,895 cases. Informed consent form was obtained from all the subjects, and the study was approved by the Local Ethics Committee of the Gregorio Marañon Hospital, Madrid, Spain, and performed in accordance with the Helsinki Declaration.

The description of the renal syndromes used as an indication for renal biopsy was previously reported^62^ and recorded as: (1) nephrotic syndrome defined as proteinuria >3.5 g/day and serum albumin levels <2.5 g/dL, (2) nephritic syndrome defined as oliguric acute kidney injury (AKI) accompanied by haematuria, proteinuria <3.5 g/day and hypertension, (3) asymptomatic urinary abnormalities (AUA) defined as proteinuria <3.5 g/day and/or haematuria and/or presence of casts, (4) AKI was defined as the increase in serum creatinine by 0.3 mg/dl within 48 hours; or the increase in serum creatinine more than 1.5 times baseline, which is known or presumed to have occurred within the prior 7 days; or urine volume lower than 0.5 ml/kg/h for 6 hours^63^, (5) chronic kidney disease (CKD) defined as abnormalities of kidney structure or function, present for 3 months, such as decreased on the estimated glomerular filtration rate (eGFR) <60 ml/min/1.73 m^2^ 64, (6) macroscopic isolated haematuria and (7) macroscopic recurrent haematuria.

The patients were categorized according to the presence of haematuria in urinary sediment, independently of the clinical manifestation, into: (1) gross haematuria (GH) when haematuria was obvious to the naked eye, (2) microscopic haematuria (mH) when the patient presented a positive dipstick, confirmed by microscopically examination, and (3) non haematuric (NH) when the sediment has <2 erythrocytes/HPF^65^. We divided follow-up into five periods of 4 years to analyze the incidence of haematuria with time. Patients were also separated into 5 groups according to age: (1) childhood and adolescence (<18 years), (2) young adults (between 19–40 year), (3) adults (between 41–65 year), (4) elderly (between 66–80 years) and (5) very elderly (>80 years). Glomerular filtration rate was estimated according to the MDRD equation^66^.

### Statistical analysis

Each year, members of the Spanish Society of Nephrology up- date common databases files (Microsoft Access^®^). Normally distributed values are expressed as mean ± SD (standard deviation), whereas abnormal variables are expressed as median ± IQR (interquartile range). The normality of distribution was analyzed by using the Kolmogorov-Smirnov test. Comparison of categorical variables was performed using the chi-square. Continuous variables were compared using the Student’s t-test for two independent samples or by analysis of variance (ANOVA) when comparing more groups. Binary logistic regression were constructed to analyse the associated risk factor for GH and mH. Statistical significance was set at p < .05. All statistical analyses were conducted using SPSS for Windows, V. 18 (SPSS^®^, Chicago, Illinois, USA).

## Additional Information

**How to cite this article**: Yuste, C. *et al.* Haematuria on the Spanish Registry of Glomerulonephritis. *Sci. Rep.* **6**, 19732; doi: 10.1038/srep19732 (2016).

## Supplementary Material

Supplementary Information

## Figures and Tables

**Table 1:** Clinical characteristic at the time of native renal biopsy according to urinalysis findings.

	All N = 19895	GH N = 1717	mH N = 10966	NH N = 7212	p
Age (years)	48.3 ± 19.5	45.8 ± 22.3	48.3 ± 18.9^a^	49.6 ± 19.0^a^^,^^b^	<0.0001
Gender (Male)	60.1%	67.2%	59.9%^a^	58.6%^a^	<0.0001
Hypertension (Yes)	53.5%	45.8%	55.5%^a^	53.7%^a^^,^^b^	<0.0001
Creatinine (mg/dL)	2.62 ± 2.84	3.32 ± 3.19	2.56 ± 2.74^a^	2.51 ± 2.75^a^	<0.0001
eGFR (mL/min)	50.4 ± 36.7	46.6 ± 41.6	50.5 ± 36	51.3 ± 36.4^b^	0.015
Proteinuria (g/day)	4.09 ± 4.61	3.09 ± 4.19	3.96 ± 4.49^a^	4.52 ± 4.83^a^^,^^b^	<0.0001

eGFR, estimated glomerular filtration rate; GH, gross haematuria; mH, microscopic haematuria; NH, non haematuria. Where ^a^ indicate statistical difference with GH, and ^b^ indicate statistical difference with mH.

**Table 2:** Relationship between urinalysis findings and kidney biopsy.

Histology	All		Gross Haematuria		Microscopic Haematuria		Non Haematuria	
	n	%	n	%	n	%	n	%
IgAN	2922	14.7	577	33.6	2029	18.5	316	4.4
Crescentic GN	2085	10.5	373	21.7	1260	11.5	452	6.3
MPGN	799	4.0	85	4.9	519	4.7	195	2.7
Mes non IgA	672	3.4	69	4	416	3.8	187	2.6
LN	1876	9.4	68	4	1110	10.1	698	9.7
FSGS	1632	8.2	66	3.8	868	7.9	698	9.7
Unclassifiable	862	4.3	62	3.6	441	4	359	5
Endocapillary	244	1.2	51	3	121	1.1	72	1
MN	2012	10.1	49	2.9	1066	9.7	897	12.5
MCD	1337	6.7	42	2.4	566	5.2	729	10.1
Amiloidosis	714	3.6	31	1.8	272	2.5	411	5.7
ATIN	611	3.1	30	1.7	217	2	364	5.1
HN	860	4.3	27	1.6	429	3.9	404	5.6
ATN	236	1.2	24	1.4	100	0.9	112	1.6
Cryoglobulinemia	111	0.6	22	1.3	65	0.6	24	0.3
TMA	144	0.7	17	1	90	0.8	37	0.5
DN	745	3.7	16	0.9	337	3.1	392	5.4
CTIN	430	2.2	16	0.9	160	1.5	254	3.5
MHT	191	1	12	0.7	104	0.9	75	1
Esclerosis	430	2.2	12	0.7	271	2.5	146	2
Fibrillary GN	43	0.2	3	0.2	20	0.2	20	0.3
MM	200	1	3	0.2	99	0.9	98	1.3
Alport S	31	0.2	2	0.1	25	0.2	4	0.1
ChE	45	0.2	1	0.1	20	0.2	24	0.3
Others	**677**	**3.4**	73	4.2	361	3.2	243	3.4
Total	**1985**	**%**	1717	8.6	10966	55.1	7212	36.3

IgAN, IgA Nephropathy; MPGN, Membranoproliferative GN; Mes non IgA, Mesangial non IgA; LN, Lupus Nephritis, FSGS, Focal Segmental Glomerulosclerosis; MN, Membranous Nephritis; MCD, Minimal Change Disease; MHT, Malignant Hypertension; ATIN, Acute Tubulointerstitial; Nephritis; ATIN, Acute Tubulointerstitial Nephritis; ATN, acute tubular Necrosis; HN, hypertensive Nephropathy; CTIN, Chronic Tubulointerstitial Nephritis; DN, Diabetic Nephropathy; TMA, Thrombotic Microangiopathy; Alport S, Alport Syndrome; ChE, Cholesterol embolism; MM, Multiple Myeloma.

**Table 3:** Top five histological diagnoses in patients presenting GH, mH and NH.

GH		mH		NH	
	%		%		%
Anti-GBM disease	29.9	IgAN	69.4	ATIN	59.6
Type 2 Crescentic	23.4	ANCA-Vasculitis	68.4	CTIN	59.1
Endocapillary	20.9	MPGN	62.3	Amyloidosis	57.7
IgAN	19.7	FSGS	53.2	MCD	54.4
Mes non IgA	10.3	MN	53	MM	53.7

Anti-GBM, anti-glomerular basement membrane, IgAN, IgA Nephropathy; MPGN, Membranoproliferative GN; Mes non IgA, Mesangial non IgA; FSGS, Focal Segmental Glomerulosclerosis; MN, Membranous Nephritis; MCD, Minimal Change Disease; ATIN, Acute Tubulointerstitial; Nephritis; ATIN, Acute Tubulointerstitial Nephritis; CTIN, Chronic Tubulointerstitial Nephritis; MM, Multiple Myeloma.

**Table 4:** Independent risk factors for haematuria in adults and paediatric patients.

	All		<18 years		>18 years	
	B	p	B	p	B	p
Independent risk factors for GH						
Age	−0.012	<0.01				
Male Gender	0.13	0.034	0.59	<0.001	0.279	<0.001
Proteinuria	−0.014	0.08	−0.13	<0.001	−0.048	<0.001
eGFR	0.002	<0.001	−0.001	0.052	−0.01	<0.001
Independent risk factors for mH						
Age	−0.007	<0.001				
Male Gender	0.08	0.049	0.058	0.66	0.06	0.047
Proteinuria	−0.026	<0.001	−0.034	0.011	−0.026	<0.001
eGFR	−0.002	<0.001	0.001	0.026	−0.001	<0.001

Binary logistic regression for the risk of present GH vs NH and mH vs NH respectively. GH, Gross Haematuria; mH, Microscopic Haematuria; NH, Non Haematuria.
