# Supplementary material for: Haematuria on the Spanish Registry of Glomerulonephritis
Source: Sci Rep. 2016 Jan 28;6:19732. doi: 10.1038/srep19732 (PMC4730139; doi:10.1038/srep19732)
Supplement: Supplementary Information [file srep19732-s1.doc]

**Supplementary from the paper:**

**HAEMATURIA ON THE SPANISH REGISTRY OF GLOMERULONEPHRITIS**

Claudia Yuste^1,*^, Francisco Rivera^2^, Juan Antonio Moreno^3,#^ and Juan Manuel López-Gómez^1,#^. On behalf of Spanish Registry of Glomerulonephritis.# Both authors share senior authorship. ^1^Gregorio Marañón Hospital, Madrid, Spain. ^2^Ciudad Real Hospital, Ciudad Renal, Spain. ^3^Renal,Vascular and Diabetes Research Lab. IIS-Fundación Jiménez Díaz. Autonóma University, Madrid, Spain.

**Supplementary Table 1.** Evolution of renal biopsies during decades according with urinalysis findings.

| **Period** | **1994-1997**  (n= 3892) | **1998-2001**  (n= 3289) | **2002-2005**  (n= 3623) | **2005-2008**  (n= 4713) | **2009-2013**  (n= 3990) |
| --- | --- | --- | --- | --- | --- |
| **Biospy number** (n)  *GH*  *mH*  *NH* | 364 (9.4%)  2300(59%)  1228(31.6%) | 272(8.2%)  1939(59%)  1078(32.8%) | 260(7%)  1986(55%)  1377(38%) | 410(9%)  2495(53%)  1808(38%) | 381(9.5%)  2038(51%)  1571(39.5%) |
| **Age** (years)  *GH**  *mH**  *NH**  *All** | 38.6±23.4  44.9±19.5  44.9±20.0  44.2±20.2 | 42.7±23.7  47.4±19.2  48.4±18.6  47.0±19.9 | 44.1±21.6  47.5±18.3  49.6±18.9  47.8±19.1 | 48.9±20.6  49.4±18.6  50.8±18.6  50.0±18.8 | 52.8±19.9  51.9±17.9  51.9±18.6  52.0±18.4 |
| **eGFR** (mL/min)  *GH**  *mH**  *NH*  *All** | 55.9±47.9  53.7±37.1  51.7±36.3  53.7±38.4 | 54.1±43.7  50.0±35.2  50.1±33.7  51.7±36.5 | 44.7±40.7  50.1±35.7  51.9±37.0  50.4±36.8 | 42.2±38.4  49.6±36.3  51.1±37.0  49.2±36.2 | 37.9±34.3  48.7±35.2  51.5±37.3  48.8±36.2 |
| **Proteinuria** (g/day) | | | | |  |
| *GH** | 2.6±3.2 | 2.7±4.1 | 3.29±4.6 | 3.3±4.3 | 3.4±4.7 |
| *mH** | 3.9±4.1 | 3.9±4.1 | 4.12±5.3 | 4.0±4.5 | 3.5±4.1 |
| *NH** | 4.6±4.4 | 5.0±5.2 | 4.9±5.1 | 4.4±5.1 | 4.0±4.2 |
| *All** | 4.1±4.1 | 4.4±4.7 | 4.4±5.3 | 4.1±4.7 | 3.7±4.2 |

eGFR, estimated glomerular filtration rate; GH, Gross Haematuria; mH, Microscopic Haematuria; NH, Non Haematuria. Where * indicates that there are statistical differences in the group between the different decades.
